# Loss of ID3 drives papillary thyroid cancer metastasis by targeting E47-mediated epithelial to mesenchymal transition

**DOI:** 10.1038/s41420-021-00614-w

**Published:** 2021-08-30

**Authors:** Sunwang Xu, Caiqin Mo, Junyu Lin, Yixing Yan, Xiaoyu Liu, Kunlin Wu, Huihao Zhang, Youzhi Zhu, Ling Chen, Xiangjin Chen

**Affiliations:** 1 Department of Thyroid and Breast Surgery, The First Affiliated Hospital of Fujian Medical University, Fuzhou, China; 2 Department of Thyroid Surgery, Quanzhou First Hospital Affiliated to Fujian Medical University, Quanzhou, China

**Keywords:** Thyroid cancer, Epithelial-mesenchymal transition

## Abstract

Papillary thyroid cancer (PTC) is the main histological type of thyroid cancer and accounts for almost all increased cases worldwide. Patients with PTC exhibit a favorable prognosis, but the fact that PTC is often accompanied by a high prevalence of lymph node metastasis (LNM) means that the overall recurrence-free survival rate in PTC patients is relatively low. Herein, we identified that ID3 expression is subdued in PTC tissues and closely associated with LNM and a poor disease-free survival outcome in PTC patients. The main contributor to this gene repression is the hypermethylation of the CpG island at the promoter of ID3. Besides, we uncovered that a loss of ID3 promotes invasion and migration of PTC cells, while an ectopic overexpression of ID3 inhibits invasion and migration. Mechanistically, ID3 exhibits tumor suppressor functions in PTC cells by interacting with E47 to form heterodimers that prevent E47 binding to CDH1 promoter and maintaining CDH1 transcription and epithelial phenotype in PTC cells. Taken together, our study demonstrates that ID3 plays a tumor suppressor role in PTC and impedes metastasis by inhibiting E47-mediated epithelial to mesenchymal transition.

## Introduction

Over the past decades, the incidence of thyroid cancer has been rapidly increasing worldwide, with the papillary thyroid cancer (PTC) subtype accounting for almost all the increased cases [1]. PTC is the major histological type of thyroid cancer, accounting for over 90% of cases. PTC derives from thyroid follicular cells and is generally iodine-avid, meaning it is amenable and can usually be cured by surgery with or without the combination of radioactive iodine ablation [2]. PTC exhibits a gentle tumor biological behavior accompanied by a low disease-specific mortality rate. However, early dissemination to local lymph nodes is quite common, leading to local or distant recurrences [3]. Cervical lymph node metastasis (LNM) is associated with an increased mortality risk in PTC patients [4]. Thus, there is an urgent need to uncover the molecular mechanisms and establish potential therapeutic targets to overcome PTC metastasis.

The inhibitor of DNA-binding (ID) proteins, including ID1–4, are a subset of helix–loop–helix (HLH) transcription factors that lack a basic DNA-binding region. ID proteins bind to basic (b) HLH E proteins to form heterodimers that cannot bind DNA or modulate transcription [5]. E proteins include E12 and E47, which are two alternative splices of the E2A gene. E47, known as a transcriptional repressor, can bind to the E-box elements within the promoter region of CDH1, inhibiting CDH1 transcription and driving the epithelial to mesenchymal transition (EMT) in various cancers [6]. As a dominant-negative regulator of E47, ID proteins dimerize with E47 and prevent its DNA interaction and transcriptional activity [7].

ID3 belongs to the ID proteins that contribute to cancer development, stemness, and metastasis [8], but depending on the cancer type. Overexpression of ID3 could maintain the stem cell features in intrahepatic cholangiocarcinoma [9] or the metastatic ability in pancreatic cancer [10]. However, the loss of ID3 could accelerate cell proliferation in lung cancer [11]. Previous studies showed that ID3 mRNA was significantly reduced in malignant thyroid lesions and PTC tissues [12, 13], but how the dysregulated expression of ID3 affects PTC cells’ biological behaviors remains poorly characterized.

In the present study, we found that ID3 transcription was significantly inhibited in PTC tissues due to hypermethylation of the CpG island at the ID3 promoter region. We further demonstrated that ID3 knockdown promotes metastatic ability in PTC cells, while overexpression of ID3 had the reverse effect. Mechanistically, ID3 interacts with E47 to prevent its binding to the CDH1 promoter to facilitate the activation of CDH1 transcription and maintain the epithelial phenotype of PTC cells.

## Results

### ID3 is downregulated in PTC and LNM tissues

**Fig. 1:**
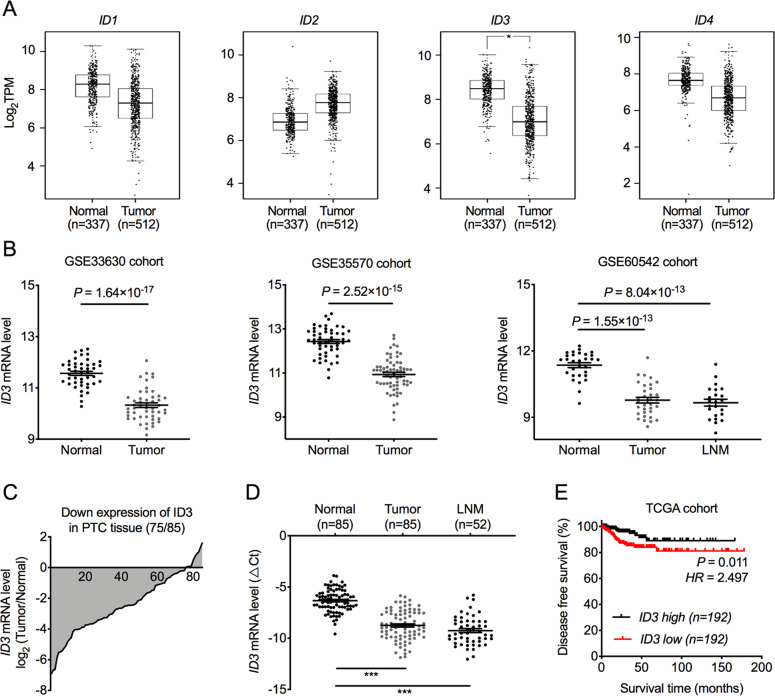
ID3 is downregulated in PTC. **A** ID1, ID2, ID3, and ID4 mRNA levels in thyroid cancer tissues and thyroid normal tissues from the TCGA and GTEx database. *P < 0.05. **B** ID3 mRNA expression levels in PTC tumorous tissues, lymph node metastasis (LNM) tissues, and adjacent normal tissues in three GEO datasets, GSE33630, GSE3557, and GSE60542. **C** The mRNA level of ID3 was measured by real-time PCR (RT-PCR) in 85 paired PTC tissues, normalized to ACTB and expressed relative to normal tissues. **D** RT-PCR results showing a significant downregulation of ID3 expression in PTC tumor tissues and LNM tissues compared to paired adjacent normal tissues. Bar, SD. ***P < 0.001. **E** Kaplan–Meier estimate of disease-free survival times in PTC patients from TCGA database according to different ID3 levels.

To characterize the expression pattern of ID proteins in PTC tissues, we first analyzed the ID1–4 expression profiles in thyroid cancer obtained from The Cancer Genome Atlas (TCGA) and The Genotype-Tissue Expression (GETx) database. The results showed that only ID3 but not ID1, ID2, or ID4 was differently expressed in tumorous thyroid tissues when compared to healthy tissues (Fig. 1A). Three GEO datasets GSE33630, GSE3557, and GSE60542 also confirmed that ID3 expression levels in PTC tumorous tissues were significantly lower than normal thyroid tissues (Fig. 1B). To further confirm the expression pattern of ID3 in PTC, we utilized real-time PCR (RT-PCR) to evaluate ID3 expression in 85 cases with paired PTC tissues. The expression level of ID3 in 88.2% of the cases (75 on 85) of PTC tissues was significantly downregulated (Fig. 1C, D). Moreover, in the GEO dataset GSE60542, ID3 expression levels in LNM tissues were also considerably lower than normal thyroid tissues (Fig. 1B). RT-PCR also confirmed that LNM tissues had lower ID3 expression than adjacent non-tumorous tissues (Fig. 1D). Besides, PTC patients with lower ID3 expression exhibited less favorable disease-free survival outcomes (Fig. 1E), suggesting that ID3 expression was highly correlated with the prognosis of PTC patients. Altogether, we demonstrate that ID3 is significantly downregulated in tumorous and LNM tissues of PTC and that ID3 inhibition is associated with PTC metastasis and poor survival outcomes.

### Promoter hypermethylation mediates ID3 silencing in PTC

**Fig. 2:**
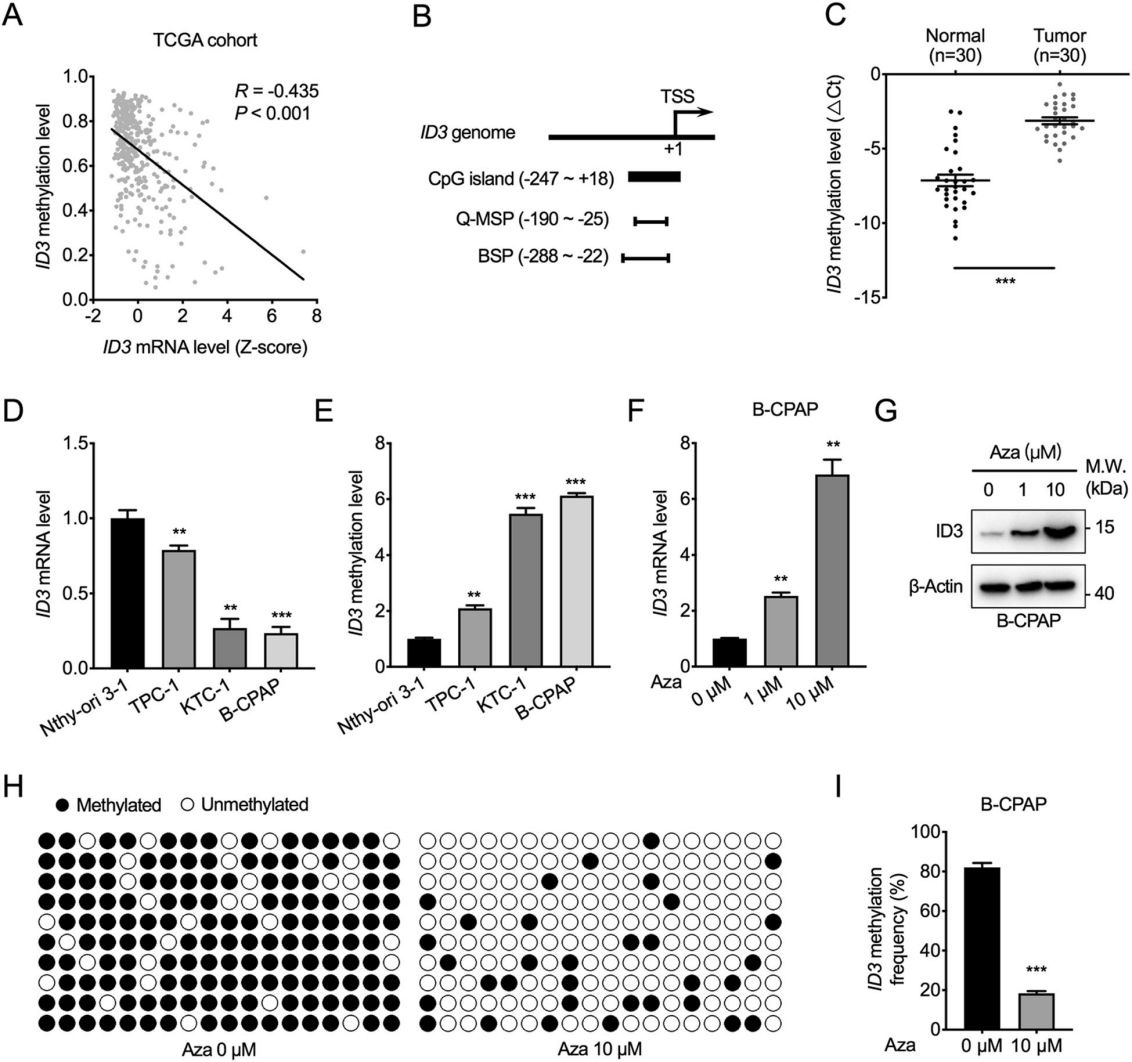
ID3 transcription is controlled by DNA methylation. **A** Pearson correlation between ID3 mRNA levels and survival years after diagnosis in patients from TCGA database. **B** The schematic diagram of the CpG island at the ID3 promoter. Q-MSP quantitative methylation-specific PCR, BSP bisulfite sequencing PCR. **C** The quantitative methylation level of the ID3 promoter was measured by Q-MSP in 30 paired PTC tissues normalized to β-actin. ΔCt = Ct_ID3_−Ct_ACTB_. Bar, SD. ***P < 0.001. **D** The mRNA level of ID3 was measured by RT-PCR in the normal thyroid cell line, Nthy-ori 3-1, and three PTC cell lines TPC-1, KTC-1, and B-CPAP. ID3 mRNA level was normalized to β-actin and expressed relative to Nthy-ori 3-1 cells. Bar, SD. **P < 0.01, ***P < 0.001. **E** The quantitative methylation levels of ID3 promoter were measured by Q-MSP in Nthy-ori 3-1, TPC-1, KTC-1, and B-CPAP cells. ID3 promoter methylation level was normalized to β-actin and expressed relative to Nthy-ori 3-1 cells. Bar, SD. **P < 0.01, ***P < 0.001. **F** The mRNA level of ID3 was measured in B-CPAP cells treated with 5-aza-2′-deoxycytidine (Aza) for 24 h and expressed relative to 0 μM. Bar, SD. **P < 0.01. **G** The protein level of ID3 was measured in B-C*P*AP cells treated with Aza for 24 h. **H** Methylation status of the ID3 promoter in B-CPAP cells treated with Aza, detected by BSP assay. Eighteen individual CpG sites within the CpG island were sequenced. open circle indicates unmethylated CpG sites and filled circle indicates methylated CpG sites. **I** The bar graph depicted the overall methylation rates of the ID3 promoter. Bar, SD. ***P < 0.001.

Transcriptional and epigenetic dysregulations are major mechanisms that regulate gene transcription activation or repression; for instance, DNA methylation of tumor suppressor genes’ promoter region can cause transcriptional silencing and promote tumor initiation and its progression [14]. Herein, we found that ID3 transcription was significantly repressed in PTC and LNM tissues. Thus, we speculated that the ID3 silencing in PTC might have resulted from DNA methylation. mRNA expression and matched DNA methylation profiles obtained from the TCGA database showed that the transcription of ID3 was significantly negatively correlated with DNA methylation of the ID3 genome (Fig. 2A). We then analyzed the CpG islands’ location within the ID3 promoter region using the MethPrimer online software [15] and localized a CpG island between −247 to +18 bp around the transcriptional start site (Fig. 2B). Quantitative methylation-specific PCR (Q-MSP) was utilized to detect and compare the relative methylation level of ID3’s CpG island within the promoter in 30 cases of paired PTC tissues. Interestingly, the results revealed that the methylation level of the ID3 promoter in tumorous tissues was significantly higher than that of normal tissues (Fig. 2C).

Besides, we found that three PTC cell lines, TPC-1, KTC-1, and B-CPAP, had lower mRNA levels but higher promoter methylation levels of ID3 than normal thyroid cell line, Nthy-ori 3-1 (Fig. 2D, E). Furthermore, treatment with the DNA methylation inhibitor 5-aza-2-deoxycytidine (Aza) in B-CPAP cells caused a reactivation of ID3 transcription in a dose-dependent manner, which was reflected by an increase in protein levels (Fig. 2F, G). Bisulfite sequencing PCR (BSP) assay further confirmed that the methylation of CpG sites within the ID3 promoter was significantly inhibited by Aza treatment in B-CPAP cells (Fig. 2H, I). The above results confirm that ID3 transcription in PTC is governed by genomic methylation status.

### ID3 inhibits the metastatic ability of PTC cells

**Fig. 3:**
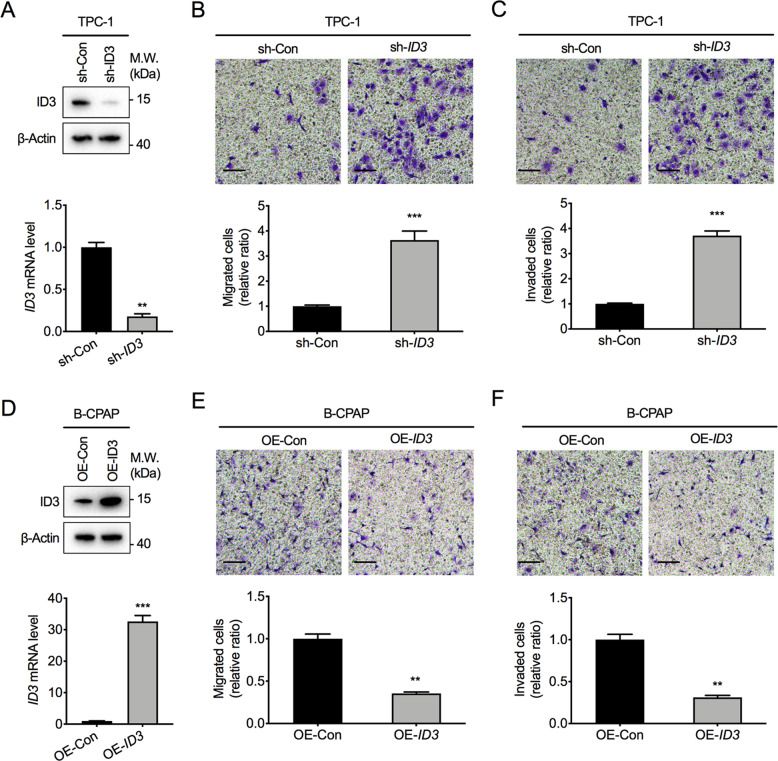
ID3 inhibits PTC cells migration and invasion. **A** ID3 protein and mRNA levels in TPC-1 cells stably transfected with ID3 shRNA (sh-ID3) or control shRNA (sh-Con). **P < 0.01. **B**, **C** Representative images (top) and relative bar graphs (bottom) depicting the relative cell migration rate (**B**) and invasion (**C**) rate normalized to control group in TPC-1 cells. Bar, SD. ***P < 0.001. Scale bars = 200 μm. **D** ID3 protein and mRNA levels in B-CPAP cells stably transfected with ID3 overexpression (OE-ID3) or control lentiviruses (OE-Con). ***P < 0.001. **E**, **F** Representative images (top) and relative bar graphs (bottom) depicting the relative cell migration (**E**) and invasion (**F**) rate normalized to control group in B-CPAP cells. Bar, SD. **P < 0.01. Scale bars = 200 μm.

We next assessed the biological effects of ID3 on PTC cells. ID3 knockdown in TPC-1 cells could significantly increase cell invasion rate and migration with or without Matrigel coating (Fig. 3A–C). In contrast, an ectopic expression of ID3 could markedly inhibit B-CPAP cells’ invasion and migration abilities (Fig. 3D–F). However, the alteration of ID3 had minimal impairment effects on cell growth for both PTC cells (Supplementary Fig. 1). These findings suggest a direct correlation between aberrant ID3 expression and the metastatic phenotype of PTC.

### ID3 interacts with E47 to maintain CDH1 expression

**Fig. 4:**
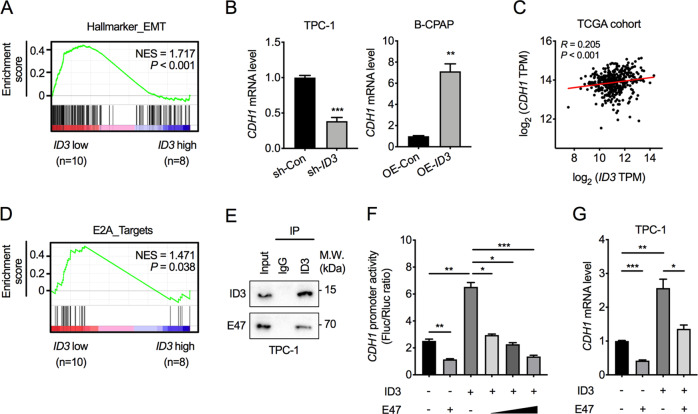
ID3 interacts with E47 to regulate CDH1 expression. **A** Gene set enrichment analysis (GSEA) result of EMT signature in ID3-low (*n* = 10) versus ID3-high (*n* = 8) expression in GSE64912 dataset. **B** CDH1 mRNA levels in ID3 knockdown TPC-1 cells or ID3 overexpression B-CPAP cells, expressed relative to corresponding control cells. Bar, SD. **P < 0.01, ***P < 0.001. **C** The Pearson correlation coefficient analysis of transcriptional levels between ID3 and CDH1 in PTC tissues from TCGA cohort. **D** GSEA result of E2A target gene signature in ID3-low (*n* = 10) versus ID3-high (*n* = 8) expression in GSE64912 dataset. **E** The interaction of endogenous ID3 protein and E47 protein was identified by immunoprecipitation. **F** The activity of CDH1 promoter affected by ID3 or E47 was measured by dual-luciferase assays. Bar, SD. *P < 0.05, **P < 0.01, ***P < 0.001. **G** CDH1 mRNA levels in TPC-1 cells transfected with ID3 and (or) E47. Bar, SD. *P < 0.05, **P < 0.01, ***P < 0.001.

To identify the differentially enriched signaling pathways in ID3-silenced PTC patients, we performed gene set enrichment analysis (GSEA) in the next-generation sequencing dataset GSE64912 of PTC tissues. The gene signature of EMT was significantly more enriched in patients with lower ID3 expression than those with higher expression (Fig. 4A). In addition, the transcription level of the E-cadherin coding gene, CDH1, was downregulated upon ID3 knockdown but upregulated when ID3 was overexpressed in PTC cells (Fig. 4B). Moreover, CDH1 expression in PTC tissues was positively correlated with ID3 in the TCGA database (Fig. 4C). These data suggest that ID3 inhibits the EMT process by regulating CDH1 expression.

GSEA results of the GSE64912 dataset also showed that the gene signature of E2A targets was further differently expressed in PTC tissues with lower and higher ID3 expression (Fig. 4D). E47 is an alternative splicing form of the E2A gene that could directly bind to the E-box motif within the CDH1 promoter to inhibit CDH1 transcription. Given that ID3 could interact with E47 to prevent E47 binding to DNA, we surmised that ID3 might interact and inhibit E47 binding to maintain CDH1 transcription in PTC cells. To that end, we performed an immunoprecipitation assay which confirmed that endogenous ID3 could interact with E47 in TPC cells (Fig. 4E). Luciferase assay further confirmed that ID3 could enhance CDH1 promoter activity, but E47 expression could counteract ID3-induced CDH1 promoter activity (Fig. 4F). We also observed that an ectopic E47 expression could counteract ID3-enhanced CDH1 transcription in TPC-1 cells (Fig. 4G).

### ID3 protein is repressed and correlated with metastatic ability in PTC tissues

**Fig. 5:**
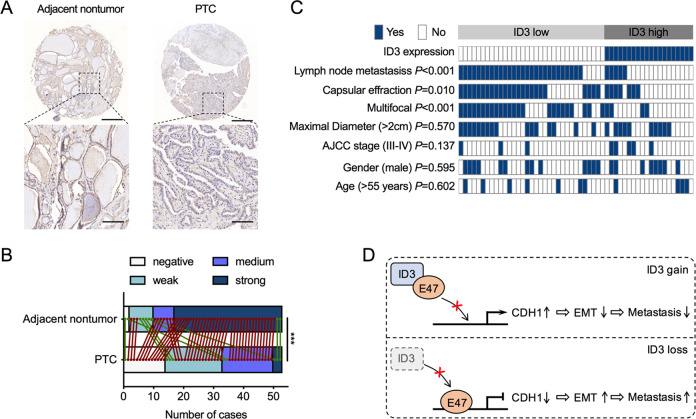
ID3 protein is repressed in PTC tissues. **A** Representative image of immunohistochemistry staining for ID3 protein on a tissue microarray constructed from 53 pairs of PTC and adjacent non-tumorous tissue. Scale bars = 500 μm (top) or 100 μm (bottom). **B** Statistical analysis of immunohistochemistry staining results for ID3 from the tissue microarray slide. **C** comparison of the lymph node metastasis, capsular effraction, multifocal lesions, maximal diameter, AJCC stage, gender, and age among 53 PTC patients according to the ID3 protein expression on a tissue microarray. **D** A schematic representation of how ID3 loss drives E47-mediated EMT in PTC cells.

Immunohistochemistry data from The Human Protein Atlas (THPA) showed strong staining of ID3 protein in the nucleus of normal thyroid glands but no staining or weak staining in PTC tissues (Supplementary Fig. 2). To confirm this result, we analyzed ID3 protein expression using a PTC tissue microarray (TMA). Consistent with the results from THPA, we found that the ID3 protein was highly expressed in 43 of 53 samples of adjacent non-tumorous tissues but lowly expressed in 32 of 53 samples of PTC tissues (Fig. 5A, B). More importantly, the expression of ID3 protein in 44 of 53 samples of PTC tissues was significantly lower than adjacent non-tumorous tissues (Fig. 5B). Finally, we analyzed the correlations between ID3 protein expression and the clinical characteristics of PTC patients. Our results showed that lower ID3 protein was significantly associated with LNM, capsular effraction, and multifocal lesions in PTC (Fig. 5C).

Altogether, our findings reveal that ID3 interacts and prevents E47 binding to the CDH1 promoter, facilitating CDH1 transcription to maintain the epithelial phenotype of PTC cells and inhibit PTC cell metastasis (Fig. 5D).

## Discussion

Although patients with PTC have excellent prognoses, with a 10-year survival rate of 80–95%, LNM commonly occurs in the early stages [16]. LNM is reported as a major risk factor for recurrence in PTC patients and is also associated with compromised survival in PTC patients [4, 17]. Recurrence in PTC patients can be fatal and impacts treatment response and survival. It is thus necessary for us to uncover the molecular mechanisms that cause PTC patients to develop LNM.

EMT is deemed to be a key process leading to functional changes in cell migration and invasion [18]. Loss of epithelial markers and gain of mesenchymal markers are two representative characteristics in the EMT process. E-cadherin is an epithelial marker that maintains cell adhesion and epithelial phenotype, and loss of this expression results in cell contact inhibition loss and cell motility increase [19]. E-cadherin expression in the tumor is controlled by various mechanisms, namely DNA methylation, EMT-associated transcription factors (EMT-TFs) inhibition, post-transcriptional regulation, and post-translational modulation. Among them, EMT-TFs, such as E47, Snail, Twist, and Zeb family, mediate the transcription inhibition of the E-cadherin coding gene, CDH1, and accelerate the EMT process [18, 20]. The present study confirmed that E47 could directly inhibit CDH1 promoter activity and repress CDH1 transcription in PTC cells. This result extends the regulatory network of E-cadherin expression in PTC cells.

It has been reported that ID3 inhibits the EMT process in non-tumorous cells [21, 22], and ID1 interplays with E47 to inhibit EMT [7], but how ID3 inhibits EMT in tumors remains unclear. Our study demonstrated that ID3 could promote CDH1 transcription and that a gradual increase in E47 ectopic expression could counteract ID3-induced CDH1 promoter activity. In addition, using immunoprecipitation, we confirmed that endogenous ID3 could interact with E47 to form an ID3–E47 complex in PTC. Because the heterodimer formed by ID3 and E47 could prevent the DNA-binding ability and transcription factor activity of E47, we concluded that ID3 inhibits the binding of E47 to the promoter region of CDH1 and prevents E47-mediated CDH1 transcription repression.

Among the four ID proteins, only ID3 was found to be dysregulated in PTC with a significantly lower expression, suggesting that ID3 might play an important role in PTC biological behaviors. Our study uncovered that the loss of ID3 enhances the metastatic ability of PTC cells, which is consistent with the results from a previous histopathological study that indicates ID3 might reduce the aggressiveness of thyroid tumors [12]. Thus, it is highly likely that ID3 works as a tumor suppressor gene in PTC metastasis. However, the tumor-suppressive effects of ID3 in tumor initiation and other malignant-related biological behaviors still require further exploration.

DNA methylation and demethylation control the transcription off and on of several mammalian cells. This is why targeting DNA methylation or demethylation has become a potential therapeutic strategy in tumors [23]. In this study, we demonstrated that the downregulation of ID3 in PTC is highly associated with hypermethylation of the CpG island in the ID3 promoter region. Importantly, DNA methylation inhibitor Aza could restore ID3 transcription in PTC cells, and ID3 overexpression inhibited PTC cells invasion and migration, suggesting that developing a DNA methylation inhibitor might be useful for targeting metastasis in PTC.

In conclusion, this study confirms that promoter hypermethylation causes the downregulation of ID3 in PTC tissues and that repressed ID3 is highly associated with LNM and poor survival outcomes of PTC patients. Suppressing ID3 promotes PTC cell invasion and migration by enhancing E47-mediated E-cadherin transcription repression and EMT progression. In light of our findings, future development of an efficient strategy to rescue ID3 expression or impede E47 DNA-binding ability could be beneficial to target PTC metastasis.

## Materials and methods

### Tissue samples

Fresh tumor tissues, LNM tissues, and adjacent nontumor tissues were collected from 85 PTC patients who received thyroidectomy and confirmed by histological examination. A TMA contains 53 PTC tissues and adjacent nontumor tissues were also collected from surgery and histologically validated. This study was approved by the Ethics Committees of The First Affiliated Hospital of Fujian Medical University, and the written informed consent was obtained from all subjects in this study.

### Cell culture

The normal thyroid cell line Nthy-ori 3-1 and PTC cell lines TPC-1, KTC-1, and B-CPAP cells were obtained from the Cell Bank of Type Culture Collection of Chinese Academy of Science, and human embryonic kidney 293T (HEK293T) cells were purchased from the American Type Culture Collection. Nthy-ori 3-1, TPC-1, and HEK293T cells were grown in high-glucose DMEM medium (Gibco, USA) and KTC-1 and B-CPAP cells were grown in RPMI-1640 medium (Gibco, USA). All cell lines were supplemented with 10% fetal bovine serum (FBS) (Gibco, USA), penicillin (100 mg/ml), and streptomycin (100 mg/ml) (Gibco, USA), and were incubated in a humidified chamber with 5% CO_2_ at 37 °C. Monthly mycoplasma tests were performed to ensure no mycoplasma contamination.

### Lentivirus infection

ID3-targted shRNA sequence (TTGTCATCTCCAACGACAA) was cloned into pGLVU6-GFP-Puro lentivirus vector, and ID3 cDNA sequence was cloned into pCDH-CMV-MCS-EF1-Puro lentivirus vector. Recombinant lentivirus particles were produced by transient transfection of HEK293T cells, along with package vectors, pMD2.G and psPAX transfected using Lipofectamine 2000 (Invitrogen, USA). The lentiviruses were harvest after transfection for 48 h. TPC-1 or B-CPAP cells were infected with lentiviruses in the presence of 10 μg/ml polybrene (Santa Cruz, USA). The infected cells were positively selected by 2.5 μg/ml puromycin (MedChemExpress, USA) to eliminate uninfected cells to generate stable cell lines.

### RNA isolation and real-time PCR (RT-PCR)

Total RNA was extracted from fresh tissues or cells using TRI Reagent (Sigma-Aldrich, USA) following the manufacturer’s protocol. One microgram of total RNA was reverse transcribed into cDNA by using 1st cDNA Synthesis Kit (Yeasen, China). RT-PCR was performed in triplicate by using SYBR Green Master Mix (Yeasen, China) and run with the Applied Biosystems ViiA TM 7 Real-Time PCR system (Applied Biosystems, USA). Data were analyzed by 2^−ΔCt^ methods and presented relative to ACTB. The RT-PCR primers for ID3 mRNA detection were ID3-RT-forward (5′–3′): CTGGACGACATGAACCACTG and ID3-RT-reverse (5′–3′): GTAGTCGATGACGCGCTGTA, CDH1 mRNA detection were CDH1-RT-forward (5′–3′): GAACGCATTGCCACATACAC and CHD1-RT-reverse (5′–3′): ATTCGGGCTTGTTGTCATTC, and ACTB mRNA detection were ACTB-RT-forward (5′–3′): CATGTACGTTGCTATCCAGGC and ACTB-RT-reverse (5′–3′): CTCCTTAATGTCACGCACGAT.

### Promoter methylation analysis

Genomic DNA was extracted from fresh tissues or cells using QIAamp DNA blood mini kit (Qiagen, GER) and dissolved in deionized water. Bisulfite treatment was performed using EZ DNA Methylation-Gold Kit (Zymo Research, USA), following the manufacture’s instructions. The Q-MSP assay was performed as described previously [24]. Briefly, bisulfite-modified DNA was subjected to hot start PCR using SYBR Green Master Mix (Yeasen, China) and run with the Applied Biosystems ViiA TM 7 Real-Time PCR system (Applied Biosystems, USA), which was initiated at 94 °C for 6 min and followed by 38 cycles of denaturation at 94 °C for 30 s, annealing at 64 °C for 25 s, extension at 72 °C for 25 s, and extra incubation at 77 °C for 30 s. Data were analyzed by 2^−ΔΔCt^ methods and presented relative to ACTB. The Q-MSP primers for ID3 promoter methylation detection were ID3-MSP-forward (5′–3′): GTTTTTTTATTTTTTGAATTCGC ID3-MSP-reverse (5′–3′): TATAAAACCTACCTAAAAAACACGC, ACTB-MSP-forward (5′–3′): TGGTGATGGAGGAGGTTTAGTAAGT ACTB-MSP-reverse (5′–3′): AACCAATAAAACCTACTCCTCCCTTAA. For BSP assay, bisulfite-modified DNA as amplified and PCR products were gel-purified and sub-cloned in to a TOPO-TA cloning vector (Yeasen, China). Ten colonies were sequenced to evaluate the degree of methylation at each CpG sites. The BSP primers for ID3 promoter methylation detection were ID3-BSP-forward (5′–3′): GGGGTGTTGTTAGGAAAAAGTAAAT ID3-BSP-reverse (5′–3′): CACTTATAAAACCTACCTAAAAAACAC.

### Migration and invasion assay

For the migration assay, the cells were starved without FBS for 4 h, then resuspended with a concentration of 50,000 cells in 100 μl medium without FBS, and cultured in the upper chamber of non-coated transwell insert (Corning, USA). In the lower chamber, 500 μl medium with 10% FBS was used as a chemoattractant to encourage cell migration. For the invasion assay, the upper chamber of the transwell inserts was pre-coated with 100 μl of 300 μg/ml Matrigel (Corning, USA), and the cells were cultured as migration assay. After 24 h incubation, all cells were stained with 0.1% crystal violet and the non-migrated or non-invaded cells were gently removed by cotton swab. The migrated or invaded cells were counted under an inverted microscope in five fields.

### Western blot

Cells were lysed in RIPA buffer containing protease inhibitor cocktail (Sigma-Aldrich, USA) and quantified with Micro BCA Protein Assay Kit (Thermo-Fisher, USA). Ten micrograms of protein were electrophoresed by 15% SDS polyacrylamide gels, transferred to PVDF membranes (Millipore, GER), and then blocked in 5% skim milk for 1 h at room temperature, incubated with primary antibodies at 4 °C overnight. Secondary antibodies were labeled with horseradish peroxidase and detected by chemi-luminescence HRP substrate kit (Millipore, GER). The antibodies used for immunoblotting were ID3 (sc-374287; Santa Cruz, USA), E47 (sc-416; Santa Cruz, USA), and β-actin (A1978; Sigma-Aldrich, USA).

### Immunoprecipitation

For the immunoprecipitation assay, cells were lysed in IP lysis buffer containing protease inhibitor cocktail (Sigma-Aldrich, USA) without SDS. The lysates were collected and immunoprecipitated with 1 μg ID3 antibody or IgG antibody at 4 °C overnight followed by incubated with Protein A agarose (Sigma-Aldrich, USA) at 4 °C for 2 h. After that, the immunocomplexes were subsequently washed with IP lysis buffer and subjected to immunoblotting.

### Luciferase assay

For luciferase assays, HEK293T cells were seed in 12-well plates at a density of 1 × 10^5^ cells per well and incubated overnight. pGL3-baisc vector containing CDH1 promoter sequences and pRL-TK vector were co-transfected with ID3, E47, or overexpression control vectors by using Lipofectamine 2000 (Invitrogen, USA). Twenty-four hours after transfection, cells were lysed, and firefly luciferase (Fluc) and renilla luciferase (Rluc) activity were determined by using the Dual-Luciferase Reporter Assay System (Promega, USA). The E-cadherin promoter activity was calculated by the ratio of Fluc to Rluc.

### Cell viability assay

For cell proliferation evaluation, cells in single-cell suspension were plated at 5000 cells in 100 µl of culture medium into 96-well plates, followed by assessment by the Cell Counting Kit-8 (Dojindo) assay every 24 h after plating. In all, 10 µl of CCK-8 solution was added to cells directly, which were then incubated at 37 °C for 1 h, followed by measurement at the absorbance at 450 nm by a Synergy 2 microplate reader (Biotek, USA)

### Colony formation assay

For colony formation, cells in single-cell suspension were plated and grown in six-well plates at a density of 100 cells per well for 14 days until colonies were visible. Later, the colonies were fixed with 4% paraformaldehyde and stained with 0.1% crystal violet.

### Immunohistochemistry analysis

For ID3 protein expression in PTC and adjacent nontumor tissues analysis, TMA slide was immunostained with anti-ID3 antibody (HPA024677; Sigma-Aldrich, USA) overnight at 4 °C at 1:200 dilution. Signal amplification and detection were performed by the DAB system according to the manufacturer’s introduction. For analysis, the stained TMA slide was photographed and converted to a digital image by a light microscope equipped with a camera (Olympus, JPN). The ID3 protein expression was determined by the evaluating staining intensity of positive staining, including negative, weak, medium, and strong.

### Statistics analysis

Data were presented as the means ± SD. Three independent repetitions were performed for each experiment. Two-tailed-unpaired Student’s *t*-tests were applied to compare the difference between two groups. Two-tailed Fisher’s exact tests were utilized to analyze the correlation between ID3 protein expression in PTC and adjacent nontumor tissues, and also clinicopathologic features. Pearson correlation coefficient was used to analyze the correlation between two parameters. The Kaplan–Meier method and log-rank test were applied to determine the disease-free survivals between different patients. All statistical calculation was performed in SPSS software (version 22.0, IBM SPSS), and a *P* < 0.05 was considered to be statistically significant.

## Supplementary information

Supplementary information

## Data Availability

The data used to support the findings of this study are available from the corresponding author upon request.
