# Supplementary material for: Loss of ID3 drives papillary thyroid cancer metastasis by targeting E47-mediated epithelial to mesenchymal transition
Source: Cell Death Discov. 2021 Aug 30;7:226. doi: 10.1038/s41420-021-00614-w (PMC8405699; doi:10.1038/s41420-021-00614-w)
Supplement: Supplementary file 1 — Supplementary information [file 41420_2021_614_MOESM1_ESM.docx]

**Supplementary information**

**Loss of ID3 drives papillary thyroid cancer metastasis by targeting E47 mediated epithelial to mesenchymal transition**

**Xu et al.**


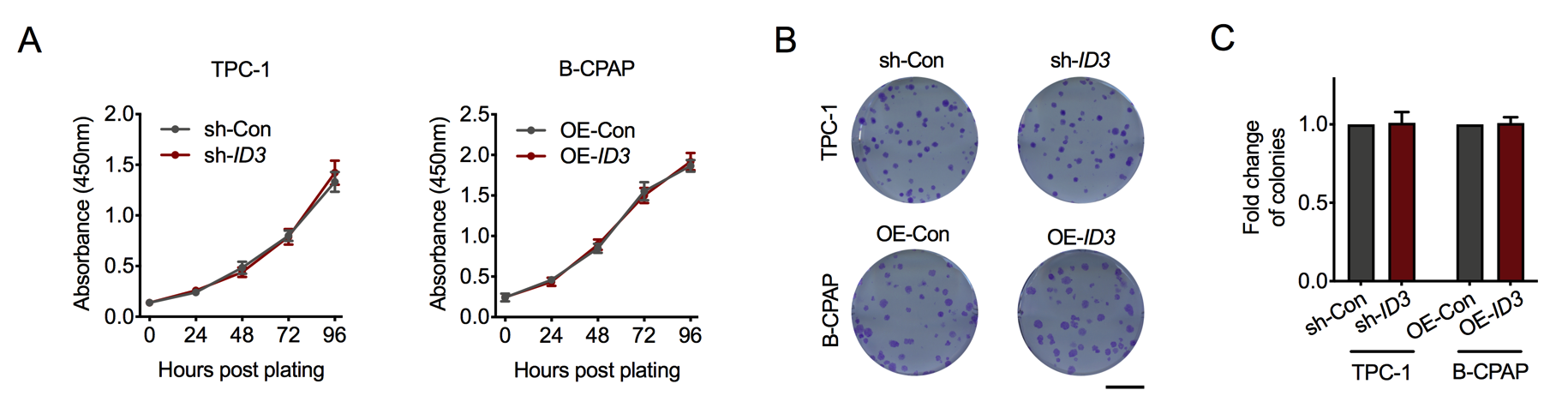


**Supplementary Fig. 1 ID3 does not affect PTC cells growth.** **A** Cell viability assays for ID3 knockdown in TPC-1 cells (left) and ID3 overexpression in B-CPAP cells (right). **B** Representative images of colony formation assays for ID3 knockdown in TPC-1 cells (left) and ID3 overexpression in B-CPAP cells (right). Scale bars = 1.0 cm. **C** Relative bar graphs depicting the relative colonies for ID3 knockdown in TPC-1 cells and ID3 overexpression in B-CPAP cells. Bar, SD.

**
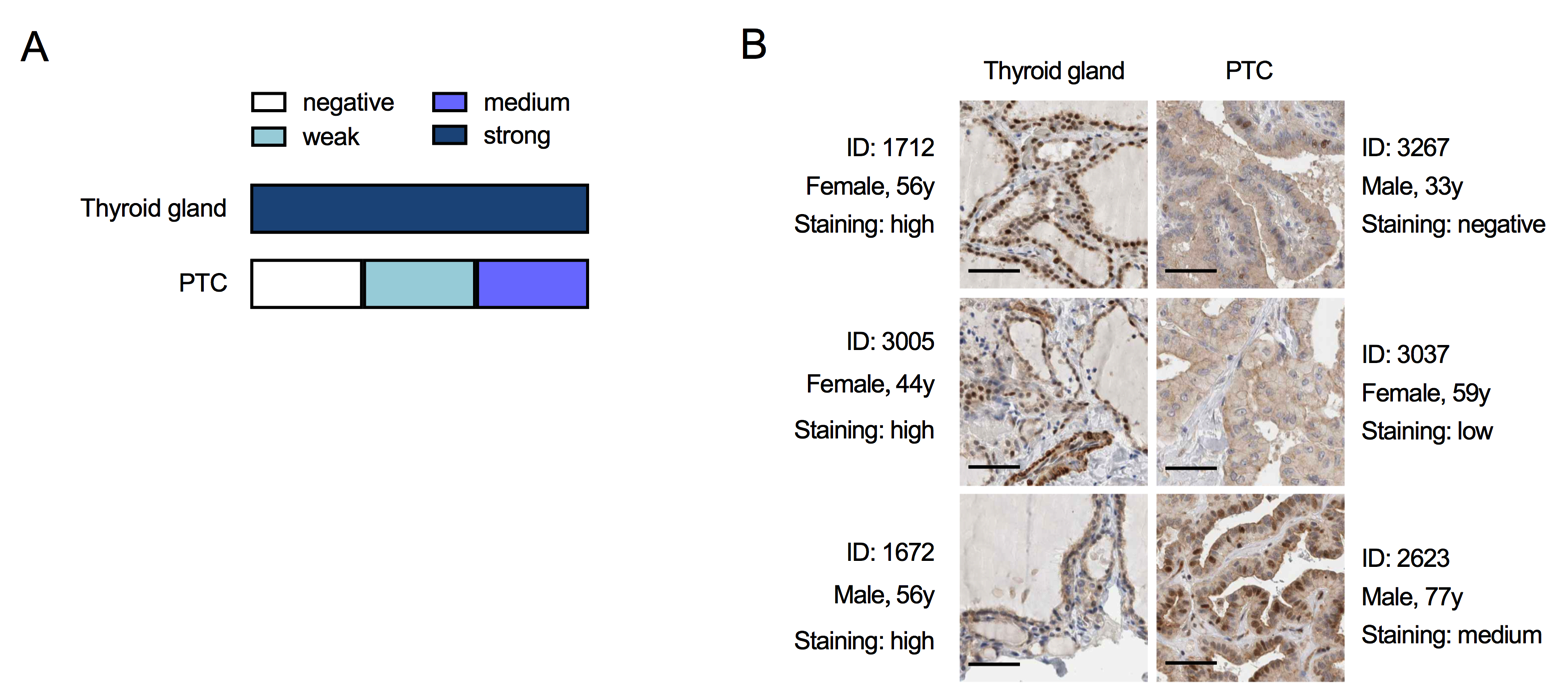
**

**Supplementary Fig. 2 Immunostaining of ID3 in PTC tissues from THPA.** **A** Immunostaining results of ID3 protein in normal thyroid glands and PTC tissues from The Human Protein Atlas (THPA). **B** Representative images of ID3 immunostaining from THPA. Scale bars = 200 μm.
